# Medical Management of Atypical Endometrial Hyperplasia: Oncological and Reproductive Outcomes at a Tertiary Center in Singapore

**DOI:** 10.7759/cureus.42685

**Published:** 2023-07-30

**Authors:** Shi Hui Lee, Carissa Ng, Pearl Wee Ling, Charissa Goh, Xiao Hui Lin, Manisha Mathur, Felicia Hui Xian Chin

**Affiliations:** 1 Department of Obstetrics and Gynaecology, Kandang Kerbau Women’s and Children’s Hospital, Singapore, SGP; 2 Department of Obstetrics and Gynaecology, Yong Loo Lin School of Medicine, National University of Singapore, Singapore, SGP; 3 Department of Reproductive Medicine, Kandang Kerbau Women’s and Children’s Hospital, Singapore, SGP; 4 Department of Gynaecological Oncology, Kandang Kerbau Women’s and Children’s Hospital, Singapore, SGP

**Keywords:** endometrial hyperplasia, endometrial cancer, progestin therapy, fertility sparing, atypical endometrial hyperplasia

## Abstract

Background

Medical management of atypical endometrial hyperplasia (AEH) includes oral or intrauterine progestins. This study aims to evaluate the oncological and reproductive outcomes of these patients and the predictive factors for disease regression, as well as to compare the treatment efficacy of different forms of progestins.

Methodology

This retrospective study was conducted at KK Women’s and Children’s Hospital, Singapore. Women diagnosed with AEH on endometrial biopsy between January 2015 to October 2017 and treated with at least eight weeks of the same progestin were included for analysis.

Results

Of the 42 patients who met the inclusion criteria, 37 were treated with oral progestins and five with the levonorgestrel intrauterine device (LNG-IUS). In total, 28 (66.6%) patients achieved complete regression (CR), but eight recurred with AEH or endometrial carcinoma. Four (9.5%) progressed to grade 1 endometrioid adenocarcinoma. Patients under 39 years old were 9.75 times more likely (95% confidence interval (CI) = 1.12-85.16, p = 0.04) to achieve CR compared to those who were 40 years old and above. In multivariate analysis, older age and higher mean body mass index had a significantly lower chance of CR. The probability of CR plateaued at nine months at 0.63 (95% CI = 0.47-0.79). There was no significant difference in time to regression, chance of regression, and risk of recurrence between oral progestin and LNG-IUS. Nine patients were trying to conceive. The clinical pregnancy rate was 44.4% (n = 4), and the live birth rate was 22.2% (n = 2).

Conclusions

Younger patients, especially those below 39 years old, are more likely to achieve CR. The value of medical treatment beyond nine months needs to be re-evaluated. There was no difference in treatment outcomes between oral progestins and LNG-IUS.

## Introduction

Endometrial cancer (EC) has been reported to be the sixth most common cancer in women [1], and the fourth leading cause of death due to gynecological cancer among women worldwide [2], making it a significant global health burden. Atypical endometrial hyperplasia (AEH) is known to have a high risk of concomitant EC [3]. The long-term risk of progression from AEH to EC has been reported to be approximately 27.5% after 19 years [4]. Although hysterectomy is currently the recommended treatment of choice [5], it is unacceptable to patients who are keen to preserve fertility or for those not fit for surgery such as those at high risk for anesthesia due to multiple medical comorbidities.

Medical treatment with systemic or local progestins has been proposed as an alternative therapy for these patients. However, there has been no consensus on the optimal treatment choice or duration for patients who are keen on medical treatment for AEH. Commonly used progestins include medroxyprogesterone acetate, megestrol acetate, and levonorgestrel intrauterine device (LNG-IUS). Meta-analyses of previous non-randomized studies have noted a high regression rate, as well as a high relapse rate with progestin treatment [6,7].

This study aims to evaluate the oncological and reproductive outcomes of patients with AEH who were treated medically and to identify the predictive factors for disease regression. The study also aims to compare the treatment efficacy with different forms of progestins.

This article was previously presented as a poster at the 2022 Annual Global Meeting of the International Gynecologic Cancer Society in New York (September 29 to October 1, 2022).

## Materials and methods

This retrospective study was conducted at KK Women’s and Children’s Hospital, a tertiary hospital in Singapore, between January 2015 and October 2017. All patients who were above 21 years of age and newly diagnosed with AEH in our center were identified from our clinical database and considered for inclusion. Those with at least eight weeks of the same progestin therapy and had at least one additional histology specimen (by office endometrial biopsy, dilatation and curettage, or hysterectomy) after completion of treatment were included for analysis. Exclusion criteria were those who had a change in the type of progestin treatment or had less than eight weeks of the same progestin therapy. All histology slides were assessed by gynecology pathologists in our institution. The patient’s demographics, medical history, type of treatment, and treatment outcomes were obtained from the hospital’s electronic medical record system. Data were collected up till the patient’s last follow-up or until July 2019, whichever was later.

Complete regression (CR) was defined as the absence of any hyperplasia or carcinoma in the biopsy sample in cases for which endometrial biopsy was performed, or in the entire endometrium in cases for which hysterectomy was done. Recurrence of disease was defined as the detection of any hyperplasia or endometrial carcinoma after CR has been achieved and before the last data collection date. Progression of disease referred to those who progressed to endometrial carcinoma before the last data collection date.

The continuous data were presented as mean ± standard deviation or median and interquartile range. The categorical data were presented as counts and percentages. Comparisons were performed using the Pearson chi-square test, Fisher’s exact test, or independent sample t-test as appropriate. The statistical significance was set at a two-sided p-value <0.05. Predictive factors for regression including age, body mass index (BMI), parity, diabetes mellitus (DM), and polycystic ovary syndrome (PCOS) were investigated with univariate and multivariate analyses using logistic regression, and odds ratios were calculated along with 95% confidence intervals (95% CIs). Plots of cumulative incidence function estimating the probability of CR over time were generated with Kaplan-Meier-One Minus Survival Function, and life table analysis was used to obtain the probability of response to compute the 95% confidence interval. All statistical analyses were performed using SPSS Version 19 software (IBM Corp., Armonk, NY, USA).

## Results

A total of 134 patients were diagnosed with AEH. Of these, 48 patients had at least eight weeks of monotherapy with progestin. Six patients were excluded as they defaulted with no repeat re-biopsy, leaving a total of 42 patients for final analysis.

The mean age at diagnosis was 41.4 ± 6.98 years old, with a mean BMI of 31.2 ± 8.39 kg/m^2^. In total, 27 patients (64.3%) were diagnosed by dilatation and curettage with hysteroscopy, and the remaining were diagnosed by bedside endometrial biopsy. The demographics and clinical presentation of our study population are presented in Table 1.

**Table 1:** Demographics and clinical presentation of the study group. Data are presented as mean ± standard deviation, median (interquartile range), or n (%).

Demographics	
Age at diagnosis (years)	41.4 ± 6.98
Gravidity	0 (0-2)
Parity	0 (0-2)
Body mass index (kg/m2)	31.2 ± 8.39
Menopaused	
Yes	3 (6.7%)
No	39 (86.7%)
Ethnicity	
Chinese	31 (73.8%)
Malay	10 (23.8%)
Others	1 (2.4%)
Clinical presentation	
Abnormal uterine bleeding or postmenopausal bleeding	37 (88.1%)
Thickened endometrium	17 (40.5%)
Endometrial/endocervical polyp	12 (28.6%)
Abnormal cervical screening test (e.g., presence of endometrial cells out of menses cycle, atypical glandular cells of uncertain significance)	4 (9.5%)
Oligomenorrhea/amenorrhea	1 (2.4%)
Subfertility workup	2 (4.8%)

Oncology outcomes

The median follow-up duration was 39 months. CR was achieved in 28 (66.6%) patients with a median time of 6.1 months (Table 2). Of these 28 patients, seven recurred with endometrial hyperplasia (25%) and one recurred with EC (3.6%). The median time to recurrence was 10.4 months from the time of regression. Four (9.5%) patients progressed to EC with a median time of 6.5 months, and all underwent surgical staging.

**Table 2:** Outcomes of medical treatment of atypical endometrial hyperplasia*. *: Another 10 patients had persistent disease at the end of the follow-up duration. †: Seven patients recurred with endometrial hyperplasia (with or without atypia), and one recurred with stage 1A grade 1 endometroid carcinoma. ‡: Median time refers to the time (months) from regression to recurrence. §: All four patients progressed to stage 1A grade 1 endometroid carcinoma. This does not include the one patient who had complete regression but recurred with endometrial carcinoma. ¶: Median time refers to time (months) from diagnosis to progression.

Outcome of treatment	Number of patients	Median time (months)
Complete regression	28/42 (66.6%)	6.1 (4.2-8.5)
Recurrence of disease	8/28 (28.6%)^†^	10.4 (4.8-17.2)^‡^
Progression	4/42 (9.5%)^§^	6.5 (3.4-14.7)^¶^

Significantly, patients who were under 39 years of age at the time of diagnosis were 9.75 times more likely (95% CI = 1.12-85.16, p = 0.04) to achieve CR compared to those who were 40 years old and above (Table 3). In the multivariate analysis, both age and BMI were found to be predictors of CR (Table 3). Older age at diagnosis was associated with a lower chance of CR (odds ratio (OR) = 0.79, 95% CI = 0.67-0.94, p = 0.01). A higher mean BMI was also found to have a lower chance of CR (OR = 0.88, 95% CI = 0.78-1.00, p = 0.05).

**Table 3:** Univariate and multivariate binary logistic regression model showing variables associated with complete regression. Data are presented as mean ± standard deviation, n, or odds ratio with 95% confidence interval.

	Patients who achieved CR (n = 28)	Patients who did not achieve CR (n = 14)	Univariate		Multivariate	
			OR (95% CI)	P-value	OR (95% CI)	P-value
Mean age at diagnosis	39.3 ± 6.5	45.4 ± 6.3	0.85 (0.75-0.97)	0.02	0.79 (0.67-0.94)	0.01
Age				0.04	-	-
<39 years old	12	1	9.75 (1.12-85.16)			
≥40 years old	16	13	Reference			
Mean body mass index (kg/m^2^)	30.0 ± 8.0	33.4 ± 9.0	0.95 (0.88-1.03)	0.23	0.88 (0.78-1.00)	0.05
Parity				0.83	1.79 (0.33-9.79)	0.50
Nulliparous	15	8	Reference			
Multiparous	13	6	1.16 (0.32-4.21)			
Diabetes mellitus				0.80	0.71 (0.11-4.41)	0.71
Yes	7	4	Reference			
No	21	10	1.20 (0.28-5.01)			
Polycystic ovarian syndrome				0.72	0.25 (0.01-5.74)	0.39
Yes	3	1	Reference			
No	25	13	0.64 (0.06-6.79)			

**Figure 1:**
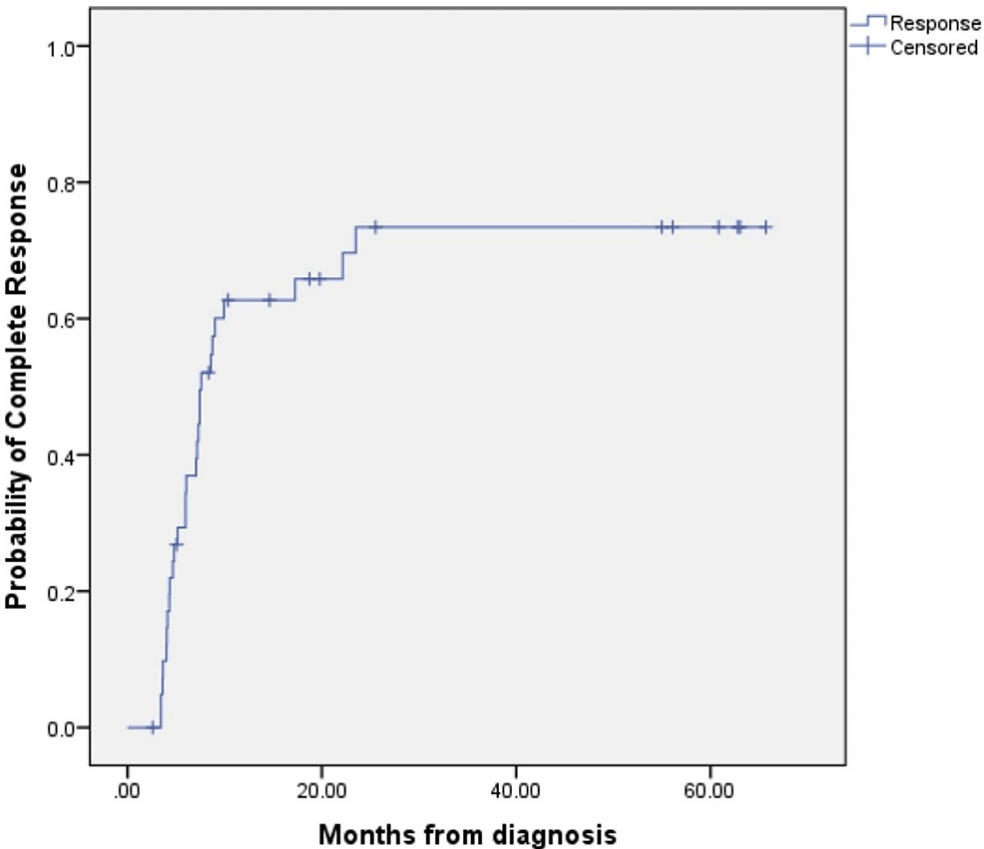
Probability of complete regression (CR) of atypical endometrial hyperplasia after the start of progestin therapy. The probability of CR over time was generated using the Kaplan-Meier-One Minus Survival Function, and life table analysis was used to obtain the probability of response to compute the 95% confidence interval. Probability of CR was estimated at 3, 6, 9, 12, 18 and 24 months intervals after the start of progestin therapy.

The probability of CR over time was estimated at three monthly intervals after the start of progestin therapy (Figure 1). The probability of CR plateaus at nine months of treatment at 0.63 (95% CI = 0.47-0.79), with a similar probability of CR from this time point onwards and even up to 18 months of treatment (0.66, 95% CI = 0.53-0.82).

Comparison of treatment efficacy: oral progestin versus levonogestrel-releasing intrauterine system

Of the 42 patients, 37 patients were treated with oral progestins, including norethisterone (20-40 mg daily), dydrogesterone (20 mg daily), and megestrol acetate (160-320 mg once daily). The other five patients were treated with the LNG-IUS. Age of diagnosis was significantly lower in patients who opted for treatment with the LNG-IUS (Table 4).

**Table 4:** Demographics of patients treated with oral progestins versus LNG-IUS. Data are presented as mean ± standard deviation, median (interquartile range), or n (%). LNG-IUS: levonorgestrel intrauterine device

	Oral progestins (n = 37)	LNG-IUS (n = 5)	P-value
Mean age of diagnosis	42.32 ± 6.65	34.20 ± 5.36	0.01
Post-menopausal	3/37 (81.1%)	0/5 (0%)	1.00
Parity	0 (0-2)	0 (0-1)	0.47
Mean body mass index (kg/m^2^)	30.44 ± 8.11	36.40 ± 9.41	0.14
Diabetes mellitus	8/37 (21.6%)	3/5 (60.0%)	0.10
Polycystic ovarian syndrome	3/37 (81.1%)	1/5 (20%)	0.41

Patients who were treated with oral progestin and those treated with LNG-IUS showed no significant difference in outcomes such as time to regression, chance of regression, and risk of recurrence (Table 5).

**Table 5:** Treatment outcomes with oral progestins versus LNG-IUS. Data are presented as mean ± standard deviation, or n (%). LNG-IUS: levonorgestrel intrauterine device; CR: complete regression

	Oral progestins (n = 37)	LNG-IUS (n = 5)	P-value
Time to CR (months)	7.81 ± 5.73	6.83 ± 2.10	0.71
Patients who achieve CR	23/37 (62.6%)	5/5 (100%)	0.15
Patients who recurred	8/23 (34.8%)	0/5 (0%)	0.28

Reproductive outcomes

Nine patients were trying to conceive. The median follow-up duration for patients who were trying to conceive was 35 months. Six patients were referred for fertility treatment but one was subsequently lost to follow up. Another three patients tried for conception naturally.

The clinical pregnancy rate was 44.4% (n = 4), of which three pregnancies were from artificial reproductive techniques (ART) and one was a spontaneous conception. The live birth rate was 22.2% (n = 2), of which one was from ART and the other from spontaneous conception.

## Discussion

Medical management of AEH achieved CR in 66.6% of our patients, but almost 30% of them had disease recurrence. These findings are similar to the current literature [6,8,9]. Our results also showed a live birth rate of 22.2%, similar to that reported in previous studies examining fertility outcomes in medically managed AEH and EC patients [6,8,10].

More importantly, our study reinforced that younger patients, especially those below 39 years old, as well as patients with lower mean BMI, are more likely to achieve CR. Previous studies have also identified younger age [11], lower BMI [12,13], and absence of PCOS [14] as positive predictive factors for CR, although these studies included a mix of patients with AEH and early-stage endometrial carcinoma. Our results may be helpful in selecting patients suitable for the medical management of AEH. It may also indicate concomitant weight loss may increase CR rates. A recent study by Chen et al did find that weight loss of ≥10% was associated with higher remission rate but this was not statistically significant (96.4% vs. 82.4%, p = 0.067) [15].

Management of AEH remains challenging given the high rates of recurrence. Literature has identified age [16], BMI [17], and diabetes mellitus [18] as significant factors for the recurrence of AEH. Due to the small number of patients who recurred in our study (n = 8), analysis for predictive factors for recurrence was not performed. It will be beneficial to investigate predictive factors for recurrence and identify methods to prevent recurrence in this population. For example, weight loss may be a useful adjunct to reduce the risk of recurrence. Chen et al. found that weight loss of ≥10% was associated with a lower recurrence rate, although this was not statistically significant (hazard ratio = 1.22, 95% CI = 0.43-3.82, p = 0.71) [15]. Recent studies investigating maintenance therapy with oral progesterone, LNG-IUS, and COC found that this may reduce the risk of recurrence of AEH [19], and may be a promising step forward for these patients.

Another controversy in the management of AEH is that the optimal duration of treatment is yet to be determined. A retrospective review found that prolongation of treatment duration from nine months to beyond nine months increased the CR rate from 76% to 95.5% in patients with AEH and EC [18]. Some authors reported that medical therapy had little benefit after 12 months if CR had not been achieved [16]. Our findings support a limited trial of medical treatment of AEH, as the probability of CR plateaus after nine months of treatment.

We did not find any difference in outcomes in terms of regression rate, recurrence rate, and time to regression with oral versus intrauterine progestins. However, it needs to be acknowledged that the sample size is limited for this subgroup analysis. Currently, LNG-IUS is recommended as first-line treatment [5], with reported higher patient compliance and satisfaction, and improved side effect profile compared to oral progesterone [20]. However, there is scanty literature comparing the effectiveness of the various medical treatments of AEH. To date, there has only been one randomized controlled trial of 19 patients with AEH in which six patients were treated with LNG-IUS and 13 with medroxyprovera acetate, concluding that there is insufficient evidence to determine if there was a significant difference between these two groups [21,22]. A previous meta-analysis with 189 AEH patients suggests a higher regression rate with LNG-IUS compared to oral progesterone (90% vs. 69%, p = 0.03). However, the authors noted that the results were derived from observational studies with mostly indirect comparisons and that it was limited by short-term follow-up in most studies [23].

Strengths and limitations

This study has several strengths. While our overall sample size is small, the majority of the current studies include women with both AEH and EC in their analysis, and our study is one of the few investigating outcomes specifically for women diagnosed with AEH. All histology slides were assessed by gynecology pathologists in our tertiary institution, where cases with challenging histopathological diagnoses are discussed in the departmental multidisciplinary team to achieve a consensus.

Several limitations need to be acknowledged. This study was conducted in a single center, which limits the generalizability of the findings. The retrospective nature of this study also would mean incomplete data such as a lack of information on compliance with medical treatment. While the median follow-up duration of 39 months in our study is comparable to other published studies, a longer duration of follow-up would be more accurate in assessing the overall oncological and reproductive outcomes. The study is also limited by possible attrition bias due to patients who were lost to follow-up during the study. Lastly, subgroup analysis comparing LNG-IUS versus oral progestin was not significant in view of the small sample size.

## Conclusions

Overall, our findings support the current understanding that medical treatment is effective, with a reasonable chance for fertility. We propose that the value of medical treatment beyond nine months should be re-evaluated. The high recurrence rate of AEH after treatment indicates that a hysterectomy should be recommended once fertility is no longer desired and long-term follow-up is otherwise needed. However, we do note that the findings of the study are limited by the retrospective nature and small sample size. Overall, patient-centered decision-making is key, and the results of this study should be used for discussion between patients and their healthcare providers. Larger prospective trials with longer follow-up periods will be helpful in determining the optimal treatment choice for AEH and evaluating fertility outcomes after medical treatment with AEH.
